# Allergens of the urushiol family promote mitochondrial dysfunction by inhibiting the electron transport at the level of cytochromes b and chemically modify cytochrome c_1_

**DOI:** 10.1186/s40659-021-00357-z

**Published:** 2021-10-28

**Authors:** Rodrigo Pacheco, Sergio A. Quezada, Alexis M. Kalergis, María Inés Becker, Jorge Ferreira, Alfredo E. De Ioannes

**Affiliations:** 1 Laboratorio de Neuroinmunología, Fundación Ciencia & Vida, Santiago, Chile; 2 Facultad de Medicina y Ciencia, Universidad San Sebastián, Santiago, Chile; 3 Cancer Immunology Unit, University College London (UCL) Cancer Institute, London, England, UK; 4 Instituto Milenio de Inmunología e Inmunoterapia, Departamento de Genética Molecular y Microbiología, Facultad de Ciencias Biológicas, Departamento de Endocrinología, Facultad de Medicina, Pontificia Universidad Católica, Santiago, Chile; 5 Fundación Ciencia y Tecnología para el Desarrollo (FUCITED), Santiago, Chile; 6 Department of Research and Development, Biosonda Corporation, Santiago, Chile; 7 Faculty of Physical and Mathematical Sciences, Department of Chemical Engineering, Biotechnology and Materials, Universidad de Chile, Santiago, Chile; 8 Faculty of Medicine, Institute of Biomedical Sciences, Molecular and Clinical Pharmacology Program, Universidad de Chile, Santiago, Chile

**Keywords:** Urushiols, Contact dermatitis, Mitochondria, Allergy, Electron transport chain, Mitochondrial respiration, Cytochrome bc1, Complex III

## Abstract

**Background:**

Urushiols are pro-electrophilic haptens that cause severe contact dermatitis mediated by CD8^+^ effector T-cells and downregulated by CD4^+^ T-cells. However, the molecular mechanism by which urushiols stimulate innate immunity in the initial stages of this allergic reaction is poorly understood. Here we explore the sub-cellular mechanisms by which urushiols initiate the allergic response.

**Results:**

Electron microscopy observations of mouse ears exposed to litreol (3-*n*-pentadecyl-10-enyl-catechol]) showed keratinocytes containing swollen mitochondria with round electron-dense inclusion bodies in the matrix. Biochemical analyses of sub-mitochondrial fractions revealed an inhibitory effect of urushiols on electron flow through the mitochondrial respiratory chain, which requires both the aliphatic and catecholic moieties of these allergens. Moreover, urushiols extracted from poison ivy/oak (mixtures of 3-*n*-pentadecyl-8,11,13 enyl/3-*n*-heptadecyl-8,11 enyl catechol) exerted a higher inhibitory effect on mitochondrial respiration than did pentadecyl catechol or litreol, indicating that the higher number of unsaturations in the aliphatic chain, stronger the allergenicity of urushiols. Furthermore, the analysis of radioactive proteins isolated from mitochondria incubated with ^3^H-litreol, indicated that this urushiol was bound to cytochrome c_1_. According to the proximity of cytochromes c_1_ and b, functional evidence indicated the site of electron flow inhibition was within complex III, in between cytochromes b_L_ (cyt b_566_) and b_H_ (cyt b_562_).

**Conclusion:**

Our data provide functional and molecular evidence indicating that the interruption of the mitochondrial electron transport chain constitutes an important mechanism by which urushiols initiates the allergic response. Thus, mitochondria may constitute a source of cellular targets for generating neoantigens involved in the T-cell mediated allergy induced by urushiols.

**Supplementary Information:**

The online version contains supplementary material available at 10.1186/s40659-021-00357-z.

## Background

Historically, contact dermatitis has been an example of specific immune reactions to modified self-antigens by a nonimmunogenic small molecule called a hapten [1]. The leaves and branches of trees belonging to the *Anacardiaceae* family, including nearly 600 species [2], e.g., poison ivy/oak, Japanese lacquer and the Chilean litre [3], among others, induce severe delayed hypersensitivity in the human skin exposed to them [4, 5]. Urushiols, the main active compounds in this reaction, constitute a family of electrophilic and lipophilic haptens that, upon oxidation, can modify skin proteins [6]. Notably, after covalent conjugation with self-peptides, haptens are recognized by T cells [1]. Similar to other contact sensitizers, urushiols induce inflammatory reactions mediated by CD8^+^ T cells [7], which secrete IFNγ [8], and are downregulated by CD4^+^ T cells in mice [7].

Until recently, it was accepted that electrophilic haptens must first modify skin proteins by attacking free amino or sulfhydryl groups of self-antigens to induce an allergic reaction [6]. Nevertheless, it was recently demonstrated [9] that unmodified urushiols from poison ivy/oak might be presented on CD1a molecules to human lymphocytes. Thus, current evidence shows two potential nonexclusive mechanisms involved in urushiol-induced allergy: the first one is associated with the presentation of unmodified urushiols as foreign immunogenic antigens, and the second one involves the generation of neoantigens by covalent modification of self-proteins [9]. Furthermore, since mice lack *Cd1a* because of a massive genetic deletion of group 2 of lipid-presenting molecules, except for *Cd1d* [10], they constitute an excellent experimental model for analyzing the role of neoantigens in allergies caused by urushiols.

Although urushiols were described at the beginning of the past century [11], little is known about the mechanisms by which the electrophilic form is acquired or the nature of their cellular targets. Similarly, the role of urushiols as activators of the innate immune response has been highlighted. Of note, activation of innate immunity plays a pivotal role in allergic responses [12], as it is necessary for the differentiation of Langerhans cells and their migration from the skin to the draining lymph nodes to trigger a secondary allergic response [13]. In this regard, the results from our previous study [7] have suggested that urushiols might generate necessary danger signaling and reactive oxygen species (ROS) [14, 15].

In the present study, we used mainly the allergen 3-(10-Z-pentadecenyl)-catechol named litreol [3], a molecule belonging to the urushiol group of allergens isolated primarily from lacquer japonica and poison oak plants (Additional file 1: Figure S1), which is obtained from *Lithraea caustica*, commonly known as the litre tree, an endemic plant of the central region of Chile. Litreol induces severe contact dermatitis in susceptible human beings [4]. Since drugs that interfere with the metabolism of fatty acids and through their uptake by mitochondria can modulate the development of allergies, our previous study was designed to show the cross talk between the extent of the allergic response to litreol and mitochondrial function [16]. Moreover, mitochondria have been considered a fundamental platform for activating innate immunity by involving a broad range of innate immune pathways, where they work as signaling platforms to intensify inflammation upon cytotoxic stimuli and contribute to effector responses [17–20]. Accordingly, we hypothesize here that mitochondria are major sources of cellular targets for generating the neoantigens involved in urushiol-induced allergy.

We used transmission electron microscopy to observ ears of BALB/c mice exposed to litreol to determine the effect at the cellular level. This initial observation revealed that mitochondria underwent dramatic structural and functional alterations. Moreover, diverse biochemical analyses of mitochondrial fractions using specific substrates and inhibitors of electron flow were used to determine the functional targets of different urushiols, including litreol, poison ivy/oak, 3-methyl catheco and 3-pentadecyl phenol. Tritium labeled litreol, 2D gel electrophoresis and mass fingerprinting were used to evaluate the ability of urushiols to chemicaly modify mitochondrial proteins. Our results reveal significan ultrastural and functional alterations of mitochondria, showing that electron transport chain is blocked by urushiols in vitro and in vivo at the level of the complex III.

## Results

### Litreol induces alterations in the mitochondrial ultrastructure

**Fig. 1:**
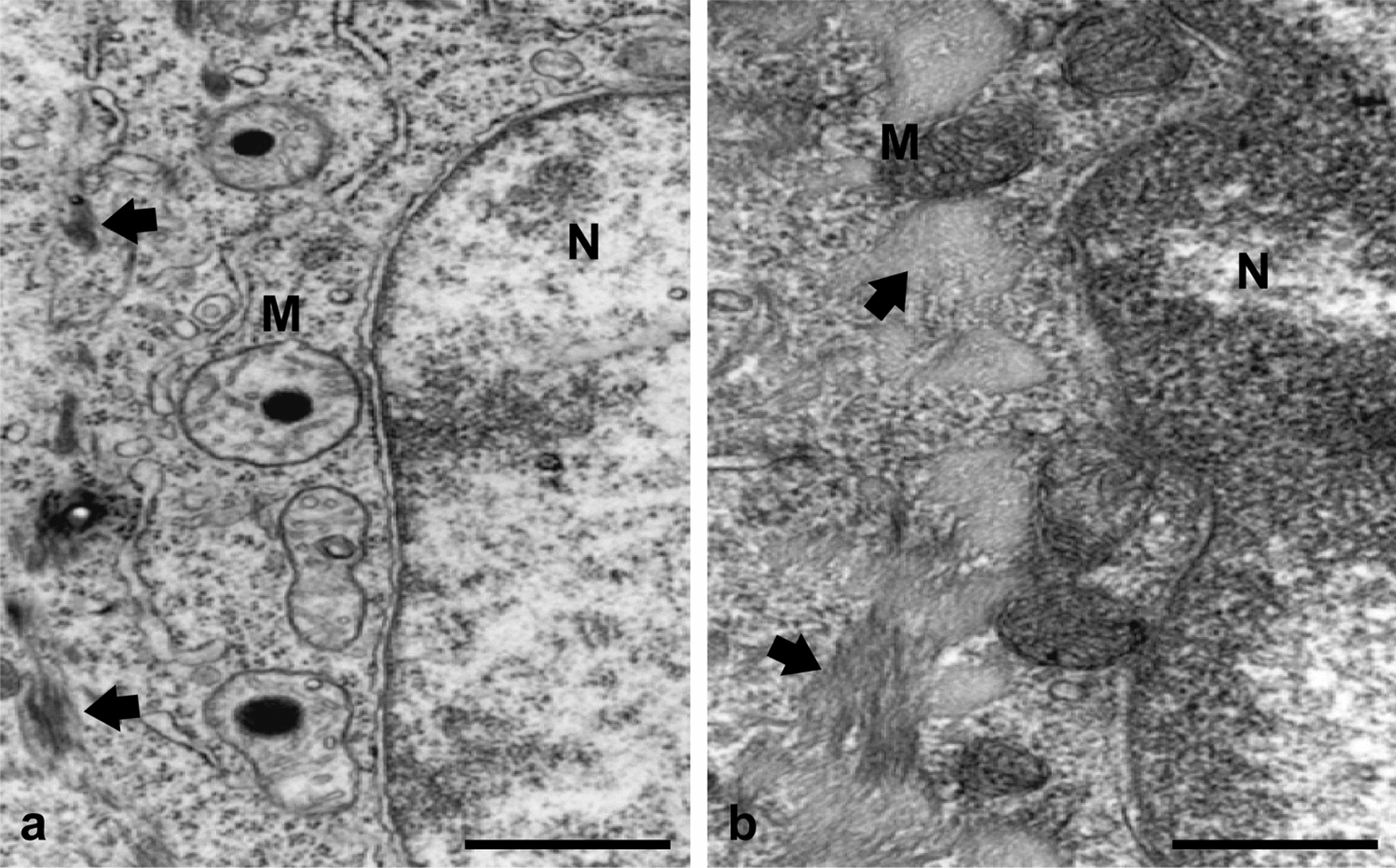
Litreol alters the mitochondrial ultrastructure. Representative transmission electron microscopy images of BALB/c mice ears: mitochondria (M) were swollen after the epicutaneous application of 1% litreol in chloroform (left) compared with the mitochondria in ears of the control mice exposed only to chloroform (right). Furthermore, round electron-dense bodies in the mitochondrial matrix, microfilaments that look disorganized (arrows), and decondensed chromatin in the nucleus (N) were observed after treatment with litreol. Bars represent 1 µm

The observation that litreol induces inflammation in SCID mice, which are devoid of an adaptive immune system, suggests that litreol can stimulate innate immunity [7]. Consequently, we investigated the effect of litreol on skin sections obtained from wild-type mouse ears after 24 h of urushiol painting. The analysis by electron transmission microscopy showed profound ultrastructural changes in keratinocytes, including chromatin decondensation and disorganization of the microfilament system (Fig. 1). The most significant observation was that the mitochondria appeared swollen, containing prominent electron-dense body inclusions in the matrix compared with the mitochondria in the control mice painted with chloroform, the litreol vehicle. To elucidate the mechanisms underlying this mitochondrial damage, we focused our study on the effect of urushiols at the level of the electron transport chain using mitochondria isolated from the rat liver, which is an extremely rich source of respiratory enzyme systems, cytochromes, and other compounds and could be analyzed with the biochemical methods used in the study [21].

### Litreol is a noncompetitive inhibitor of complex III at the level of the cytochrome bc_1_

**Fig. 2:**
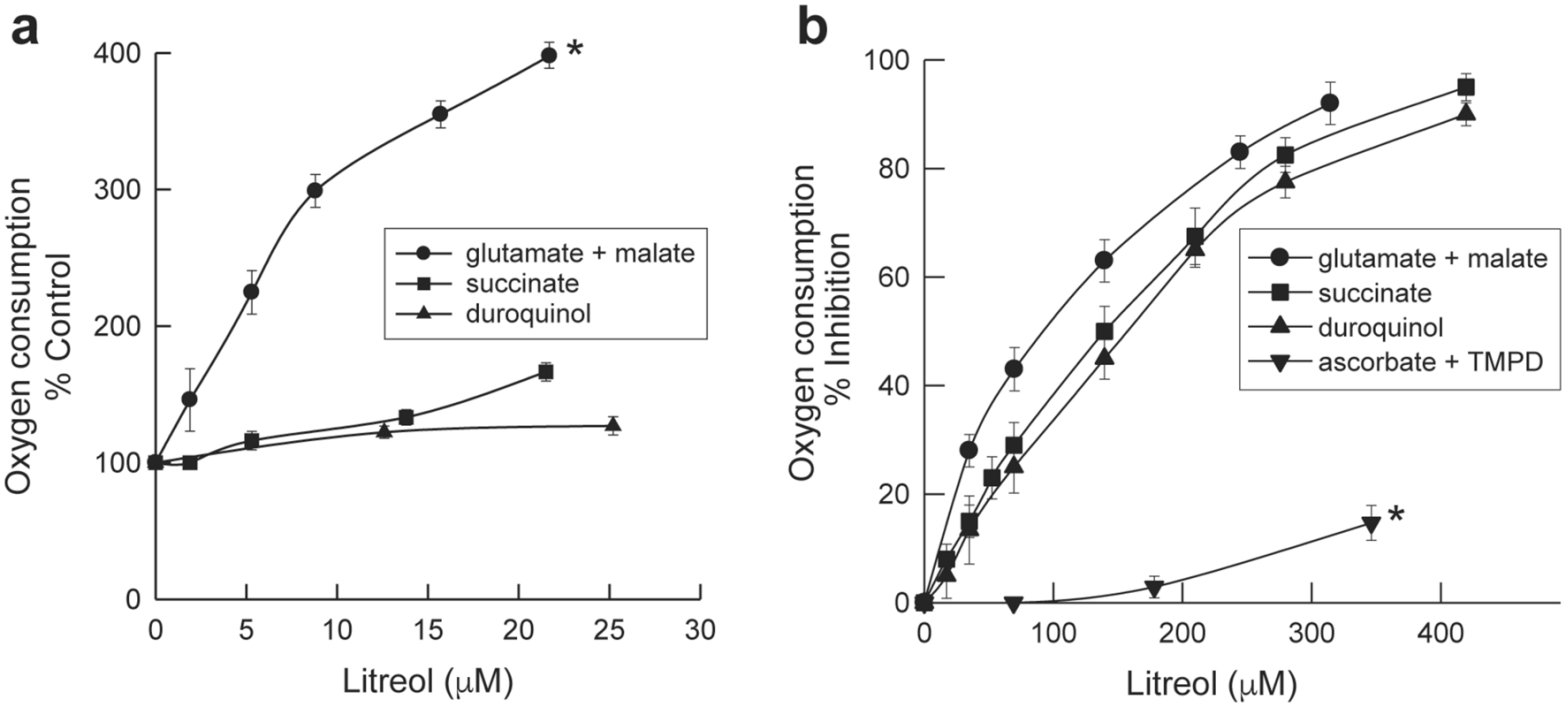
Effect of urushiol on the respiration of rat liver mitochondria. Time course rates of oxygen consumption were measured by polarography with a Clark oxygen electrode. The percentage of inhibited oxygen consumption stimulated by different respiration substrates in the presence of CCCP and increasing concentrations of litreol in DMSO was determined as described in the “Methods” section. **a** Effect of litreol on the resting state of respiration (state IV) of rat liver mitochondria. Control rates for glutamate + malate (black circle), succinate (black square), and duroquinol (black up-pointing triangle) oxidation were 8.9, 26.9, and 54.7 nmoles O/min/mg protein, respectively. Results are expressed as the mean percentage of the control ± SD of four independent experiments. Glutamate + malate curve vs succinate or duroquinol curves: p < 0.05; succinate vs duroquinol curves: not significant. **b** Litreol inhibits respiration at the level of complex III in isolated rat liver mitochondria. Control rates for CCCP-stimulated glutamate + malate (black circle), succinate (black square), duroquinol (black up-pointing triangle) and ascorbate + TMPD (black down-pointing triangle) oxidation were 65.4, 109.9, 163.9 and 214.8 nmol O/min/mg protein, respectively, in the presence of 0.02 μM CCCP. Results are expressed as the mean percentage of the control ± SD of three independent experiments. Ascorbate + CMP curve vs curves of other compounds: p < 0.05. Glutamate + malate curve versus succinate or duroquinol curve: not significant

Next, to define whether urushiols exerted an inhibitory action along the respiratory chain, we first studied the effect of urushiol on the respiration of rat liver mitochondria in a resting state. As expected, litreol stimulates a resting state of oxygen consumption at the level of complex I due to its redox properties (Fig. 2a). Then, we investigated the effect of litreol on oxygen consumption rates after adding different electron donors in the presence of carbonyl cyanide *m*-chlorophenylhydrazone (CCCP) (Fig. 2b). The results showed that only *N*,*N*,*N*ʹ,*N*ʹ-tetramethyl-*p*-phenylenediamine (TMPD) plus ascorbate an electron donor to complex IV, was able to overcome the inhibition of mitochondrial respiration induced by litreol. Furthermore, we observed similar hyperbolic inhibitory curves when the effects of urushiols and those of known complex III inhibitors were compared, thus suggesting that complex I and II were not involved. Thus, taken together, these results indicate that the inhibition to electron flow induced by urushiols seemed to be restricted at the cytochrome bc1 level.

**Fig. 3:**
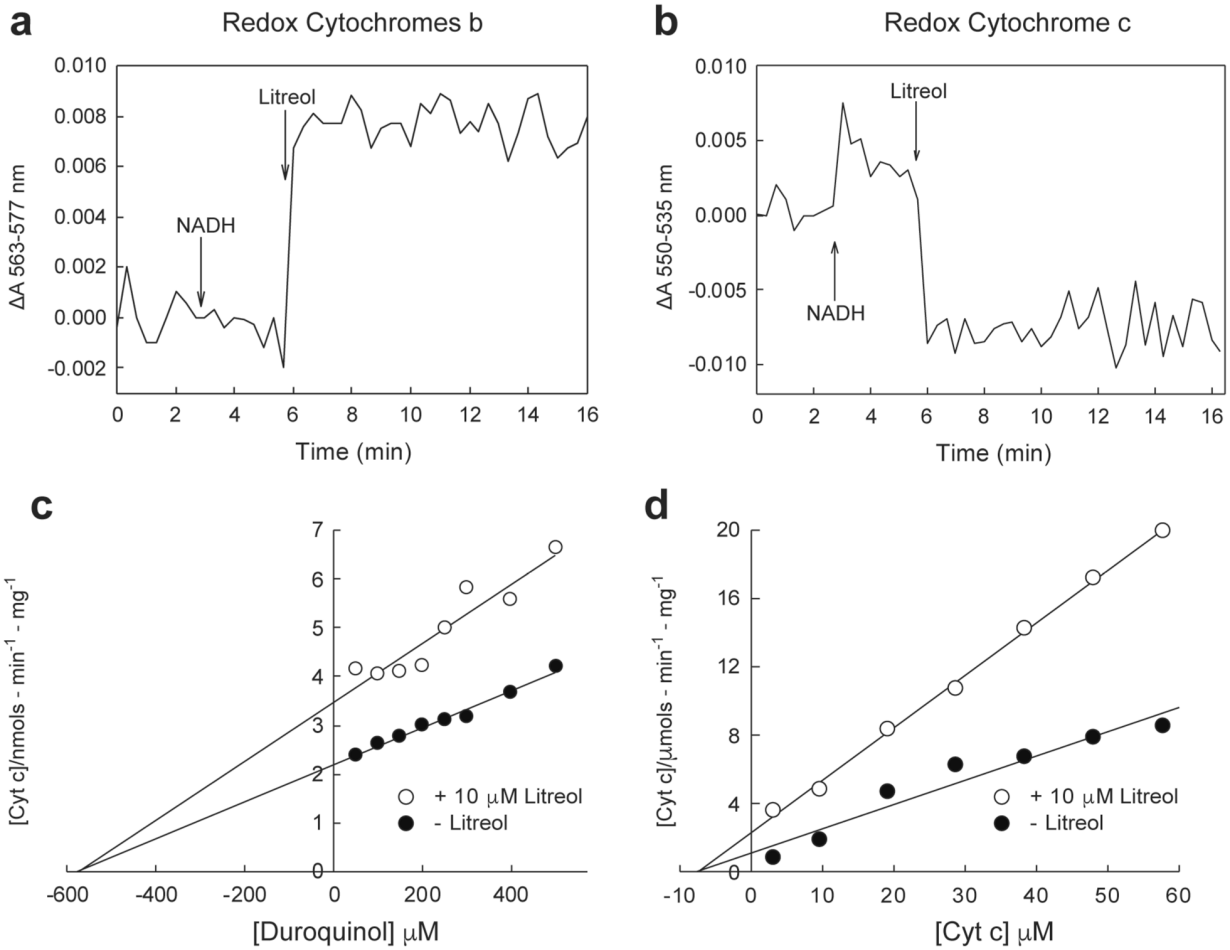
The inhibition of the electron flow in complex III by litreol involves cytochrome b. Reduction in cytochromes b and c_1_ was measured by spectrophotometry in submitochondrial particles and cytochrome c, as described in “Methods” section. The reaction was initiated by adding 0.17 mM NADH and was monitored at 563–577 nm for cytochrome b (**a**) and 550–535 nm for cytochrome c (**b**). The time of each cycle was 20 s, and the reading time was 0.5 s. NADH and litreol were added after 9 and 18 cycles, respectively, as indicated by the respective arrows; in total, 50 cycles were completed. The results are representative of four independent experiments. **c**, **d** Hanes-Woolf kinetics of the reduction of cyt c in the presence of litreol and antimycin A. Duroquinol and cyt c were used respectively as electron donor and electron acceptor. The results from the Hanes-Woolf analysis indicate that the inhibition was noncompetitive for cyt c

To define the effect of litreol on the electron flow within complex III, the redox status of both cytochromes b and c was determined by spectrophotometry after the addition of NADH as the substrate in the presence of litreol. As shown in Fig. 3, both b cytochromes were reduced after adding litreol (Fig. 3a), whereas cytochrome c (Fig. 3b) remained oxidized, suggesting that the interruption of electron flow affected one or both b cytochromes.

Complex III is present in the mitochondrial inner membrane and catalyzes electron transport from ubiquinol (QH2) to cytochrome c, coupling the translocation of protons across the membrane [22]. The complex contains ubiquinol (electron donor)-binding site and a cytochrome c (electron acceptor)-binding site. Thus, to understand the mechanisms of litreol inhibition of complex III, we tested whether litreol interferes with the binding of the electron donor duroquinol (Dur) and the electron acceptor cytochrome c. The enzyme kinetics of complex III for Dur and cytochrome c were measured at litreol concentrations of 10 µM. The results (Fig. 3c, d, respectively) from the Hanes-Woolf analysis showed that the inhibition of electron flow through the mitochondrial transport chain was dependent on either the concentration of Dur or cytochrome c, indicating noncompetitive kinetics.

### Litreol halts the electron flow between cytochromes b_L_ and b_H_

**Fig. 4:**
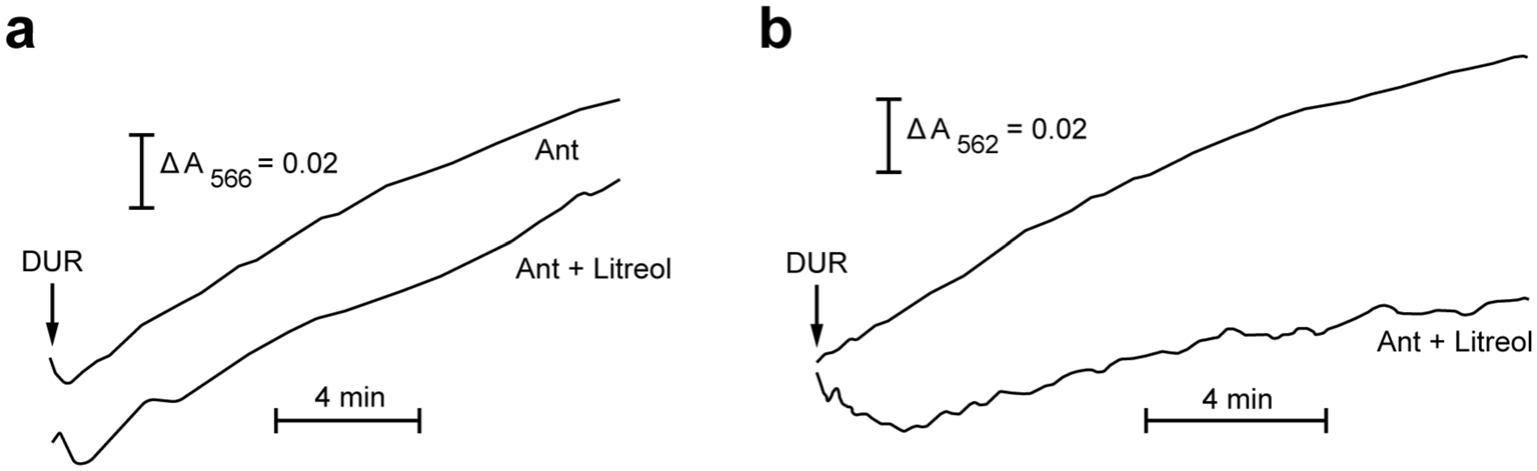
The effect of litreol on the redox states of cytochromes b_566_ and b_562_ in rat liver submitochondrial particles indicating that the electron flux is halted in between cytochromes b. Two milliliters of the reaction mixture were immediately stirred manually after each addition during the automatic wavelength change. **a** The antimycin-sensitive pre-steady-state reduction kinetics of cytochromes b_566_ and **b** b_562_ were monitored in submitochondrial particles dissolved in 1% DM of the 1.0 ml reaction medium. Ant, 5 µM antimycin A presence only; Ant + Litreol 5 µM antimycin A and 10 µM litreol. For other experimental conditions, see the “Methods” section. The results are representative of three independent experiments

To gain deeper insight into the mechanism underlying urushiol-mediated inhibition of mitochondrial respiration, we investigated the effect of litreol on electron flow through both b cytochromes. Accordingly, antimycin A (Ant), as a Q_i_-specific inhibitor, slowed electron flow. Figure 4 shows the redox status of cytochrome b_L_ (cyt b_566_) and cytochrome b_H_ (cyt b_562_) using Dur as the substrate in the presence of 0.1% dodecyl maltoside. The results show that, when litreol was added, cyt b_566_ was reduced (Fig. 4a), while cyt b_562_ remained almost totally oxidized (Fig. 4b), indicating that the electron flow was halted only at the level of cyt b_566_.

### Both the catecholic moiety and the aliphatic chain are needed for litreol to inhibit the flow of electrons in complex III

**Fig. 5:**
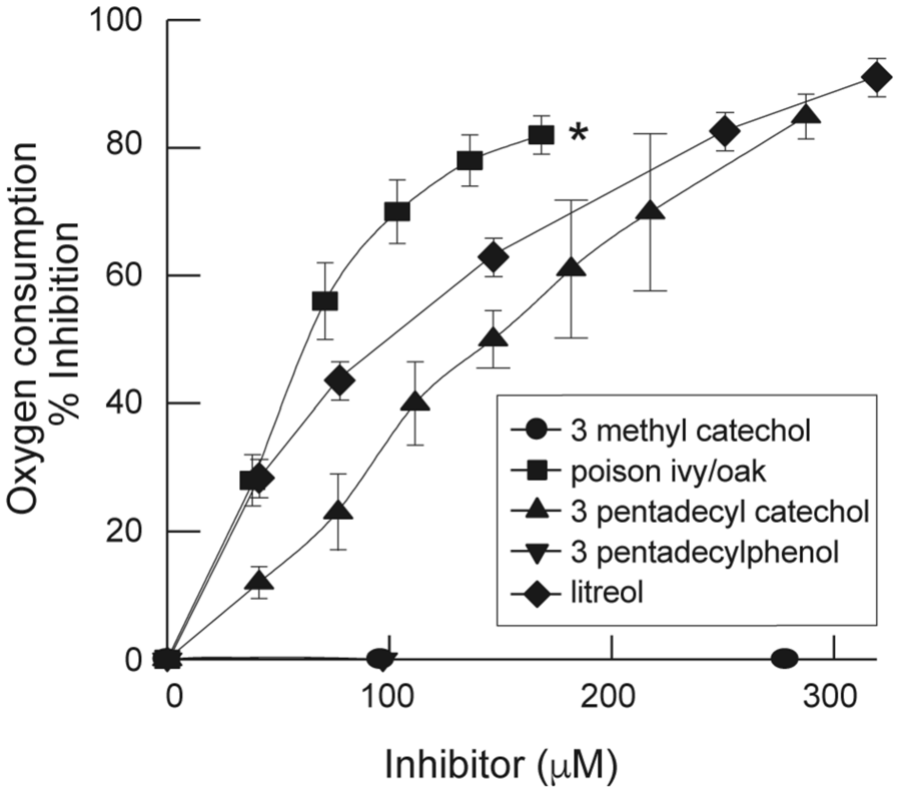
Both catecholic and aliphatic moieties are required for the inhibition of coupled respiration. The effects of litreol, poison oak, and compounds structurally related to urushiols on mitochondrial respiration were compared using 2.8 mM glutamate-malate as a substrate for respiration and 0.28 ADP as the initiator of the reaction. The control rate for ADP-stimulated glutamate + malate oxidation was 66.0 nmol O/min/mg protein in the presence of 0.28 mM ADP. Poison ivy/oak (black square), 3-pentadecyl catechol (black up-pointing triangle), 3-methyl catechol (black circle), 3-pentadecyl phenol (black down-pointing triangle), and litreol (black diamond suit). Poison ivy/oak vs pentadecyl catechol or litreol: p < 0.05; litreol vs pentadecyl catechol: not significant

To determine the minimal structural requirements of urushiols to inhibit respiration, we compared the inhibitory effect of poison ivy/oak, pentadecyl catechol, and two non-urushiol analog compounds, pentadecyl phenol and water-soluble 3-methyl catechol, on rat liver-isolated mitochondria. Only at very high concentrations, the two non-urushiol compounds did exert marginal inhibition. Among the urushiols, poison ivy/oak had the most potent effect (Fig. 5), thus suggesting that the increase in double bonds along the aliphatic chain (see urushiols structures at Additional file 1: Figure S1) intensified the allergenicity of urushiols. All the inhibitor molecules, including litreol, were catecholic in nature, and they each have both catechol and aliphatic moieties.

### Cytochrome c_1_ is chemically modified by litreol

**Fig. 6:**
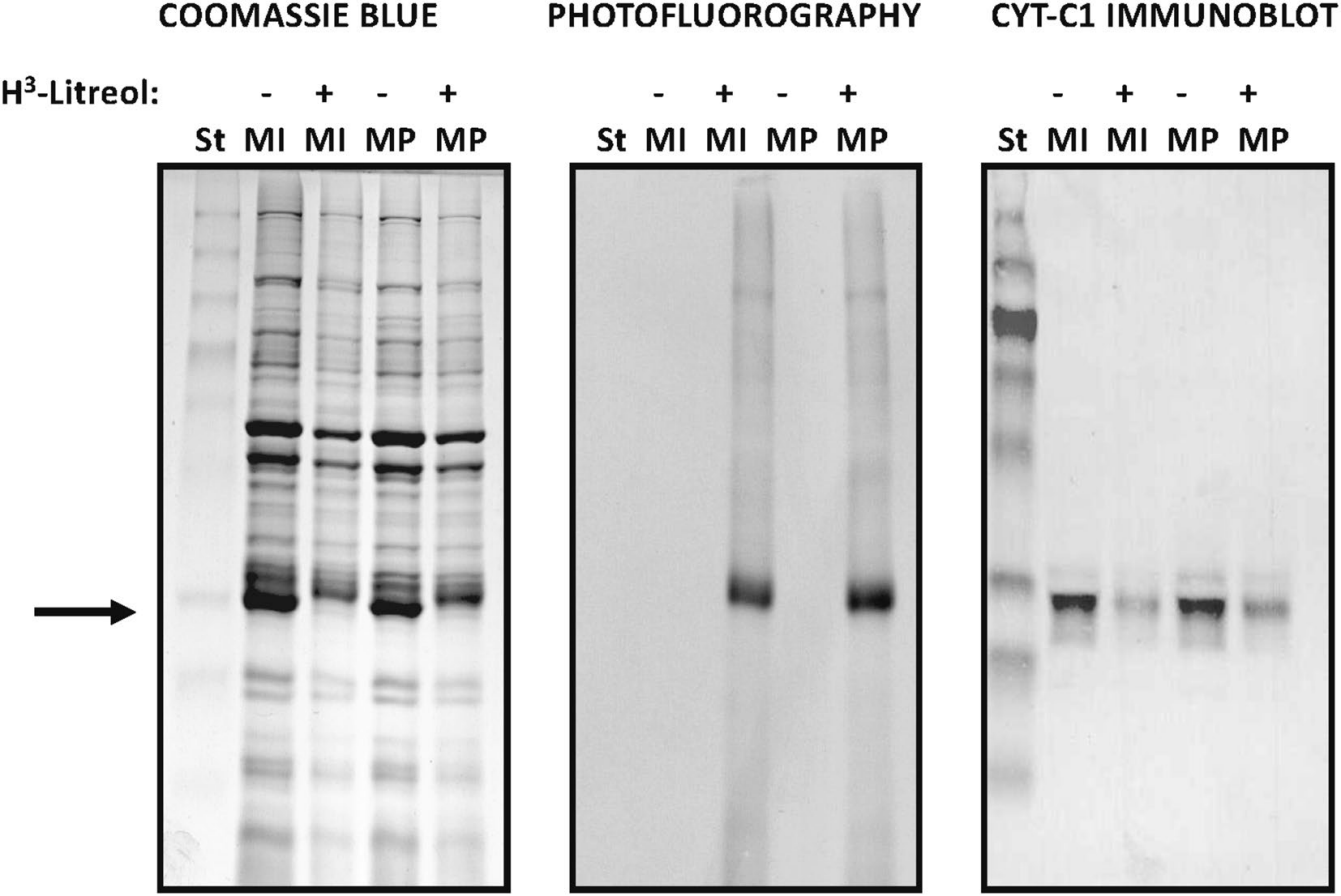
Litreol binds to an inner mitochondrial membrane protein from bovine heart mitochondria with a molecular weight of approximately 27 kD. Mitochondria isolated from the bovine heart were treated with or without 5 μM ^3^H-litreol and then with cold litreol, according to the “Methods” section. After submitochondrial fractionation, the proteins of the internal membranes (IM) and mitoplasts (MP) were analyzed by SDS-PAGE. The fractions from mitochondria treated with ^3^H-litreol are indicated. Two identical gels were generated: one was stained with Coomassie blue (left panel) and then subjected to fluorography (middle panel). The proteins in the other gel were transferred to a nitrocellulose membrane and developed by Western blotting using an anti-cytochrome c_1_ antiserum (right panel). A radioactive band (arrow) was observed in the mitoplast and inner membrane fractions and showed electrophoretic mobility similar to that of cytochrome c_1_ (27 kD)_._ St: prestained marker

**Fig. 7:**
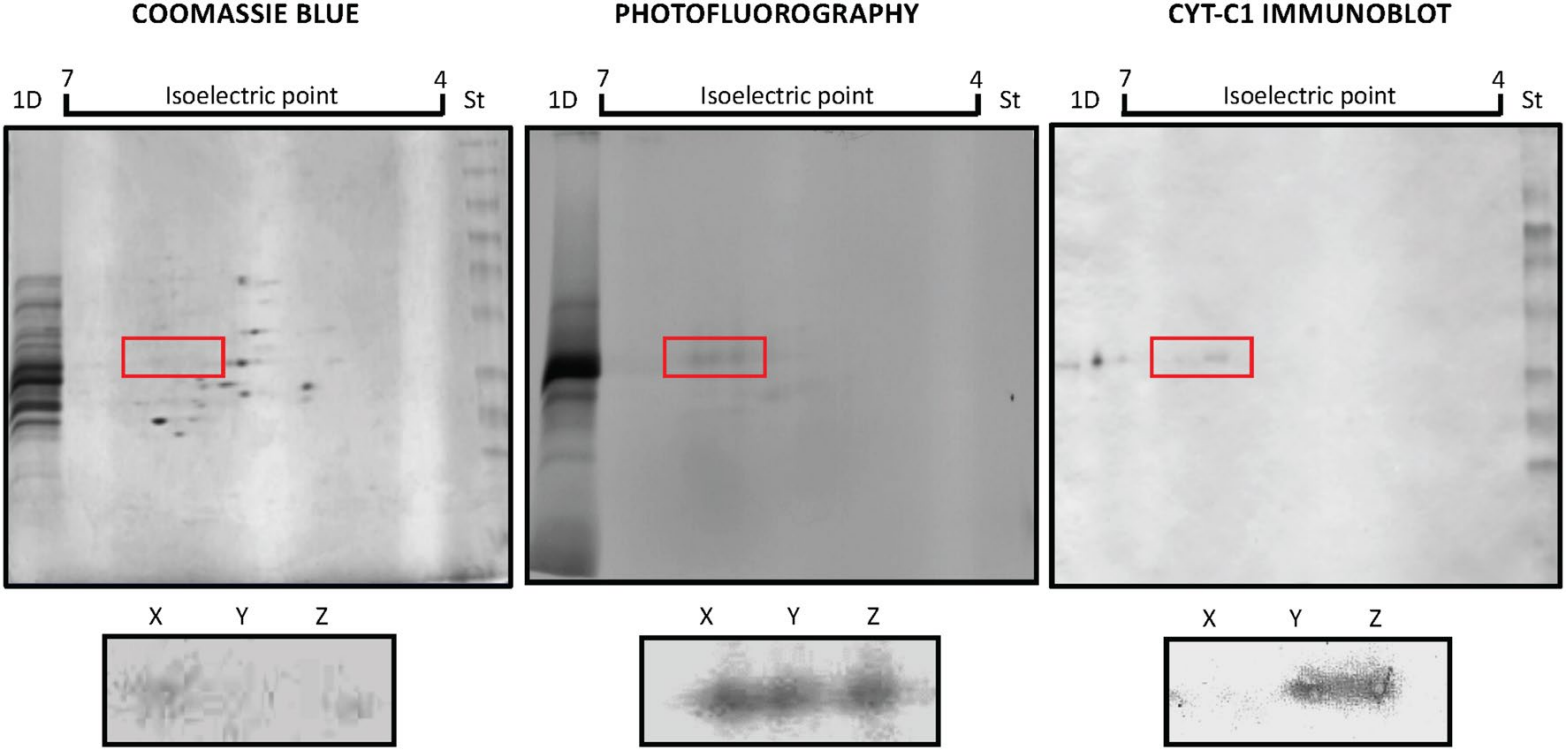
Litreol modifies the isoelectric point and immunoreactivity of cytochrome c_1_. 100 μg of protein extracted from the inner membrane from bovine heart mitochondria were separated by 2D-PAGE gels, and then protein was detected with Coomassie blue staining (left panel), radioactivity was assessed with photofluorography (middle panel), and cytochrome c_1_ immunoreactivity was evaluated by western blot (right panel). A magnification of the area indicated in red is shown below each panel. X, Y and Z represent the 27 kD radioactive spots with isoelectric points of 6.35, 6.19, and 6.02, respectively. 1D indicates a lane where 100 μg of protein extracts were separated only by SDS PAGE. St indicates a lane where molecular weight standards were loaded in the SDS PAGE. The gradient of pH generated (isoelectric point) is indicated

Since urushiols might have stopped mitochondrial respiration by directly modifying cytochrome b or altering a different molecule related functionally or structurally to cytochrome b, we next sought to identify the molecular target of litreol. For this purpose, we inhibited the electron chain using ^3^H-labeled pentadecyl catechol in mitochondria isolated from bovine hearts, and then we analyzed the ^3^H-labeled proteins by SDS-PAGE stained with Coomassie blue and then by photofluorography (Fig. 6). We observed the preferential radiolabeling of a molecule of approximately 27 kD in the mitoplast and in the inner membrane fractions (Fig. 6). Moreover, the radioactive band overlapped with that of cyt c_1_ in the Western blots (Fig. 6). Since the mobility of cyt c_1_ and cyt b is similar on the one-dimensional SDS gels [23], we sought to analyze more precisely the molecular target of litreol using two-dimensional gel electrophoresis (2D-PAGE) [24]. For this purpose, the radioactive fraction of proteins obtained from the inner membrane of bovine heart mitochondria were enriched by molecular exclusion using a Sephadex G200 column and then separated by 2D-PAGE gels. The photofluorography analysis of proteins separated by 2D-PAGE gels revealed three spots of proteins of 27 KD with different isoelectric points; 6.35, 6.19 and 6.02, respectively (Fig. 7, middle panel). Interestingly, the Coomasie blue staining detected only the spot with isoelectric point 6.35 (Fig. 7, left panel), suggesting that this protein was more abundant than proteins with isoelectric points 6.19 y 6.02. Moreover, the western blot analysis of 2D-PAGE gels revealed that proteins with isoelectric points 6.19 y 6.02 present immunoreactivity with the anti-cytochrome c_1_ antibody (Fig. 7, right panel). Considering the aminoacidic sequence of the cytochrome c_1_ (Additional file 1: Figure S2) and the lipophilic nature of litreol, these results suggest that this allergen modifies the lysine residues of cytochrome c_1_ located inside of or close to the inner membrane (Additional file 1: Figure S2), thus neutralizing the positive charge of the ε-amino groups and thereby shifting the isoelectric point of the protein. The absence of a spot with isolectric point of 6.35 in the immunoblot for cytochrome c_1_ (Fig. 7, right panel, spot X) suggests that the adduct of litreol-cytochrome c_1_ that results in a protein with isolectric point of 6.35 loses the immunoreactivity to the antibody used for westernblot analysis due to the distortion of immunodominant epitopes. Alternatively, it could be explained by the fact that some blocking agent used for immunoblots might mask some specific epitopes, thereby preventing antibody binding, such as described for milk. To complement the analyses of identity of the protein modified by litreol, we performed a mass fingerprinting analysis on both radioactive spots in slices excised from the 2D-PAGE SDS slab gel (data not shown). After trypsin in-gel digestion, PAGE was run in parallel with unmodified cyt c_1_ (control), and four tryptic fragments assignable to cyt c_1_ were observed at the same spots in both gels (1.246, 1.670, 1.819 and 1.863 kD) (Additional file 1: Figure S3a). However, the radioactive spots from both urushiol-treated samples indicated only two peptides, with sizes of 1.670 and 1.863 kD (Additional file 1: Figure S3b, c). One of the missing fragments likely corresponds to the amino-terminal fragment 1–15 (1.819 kD), SDLELHPPSYPWSHR, on the amino terminus that would be expected to be 2.139 kD after the addition of litreol. The other missing fragment (192–202) of 1.246 kD, WAAEPEHDHRK, is close to the transmembrane region, and after litreol addition, its size would be greater than 2.120 kD. Nevertheless, it was also expected that, after the addition of litreol, proteolytic cleavage at lys 202 would be impaired; therefore, the size of the resulting peptide would likely be greater than 2.279 kD. In both cases, the radioactive fragments are outside the detection limit of the mass spectrometer (Additional file 1: Figure S3). Together, these results strongly suggest that cytochrome c_1_ is the molecular target of litreol and that the addition of urushiol impairs electron transport through complex III (between cyt b_L_ and cyt b_H_).

## Discussion

Skin is an essential physical and biological barrier protecting from the penetration of pathogenic microorganisms, and at the same time, it allows gas exchange and respiration and is a substrate for the development of beneficial microbiota [25]. However, the hydrophobic nature of the external layer allows the absorption of natural and manufactured lipidic molecules that are absorbed and can diffuse deep into the epidermis membranes, causing unwanted inflammatory reactions, including contact dermatitis [26]. Between the external coating and the germinal epithelium, a sheet of keratinocytes and Langerhans cells, among other cells, serves as an immune battlefield for lymphocytes coming from capillaries to attack foreign antigens, including lipophilic bacterial constituents from mycobacteria described for the first time by Porcelli et al. [27] and self-modified proteins by haptens [28]. Of note, urushiols are among the most potent allergens to humans because of their lipophilicity and reactive catechol moiety [9].

It is well known that mitochondrial functions are intrinsically linked to their morphology [29]. Thus, from the ultrastructural changes observed in the mitochondria following the exposure of keratinocytes to litreol, we show in this study that the inhibition of mouse mitochondrial respiration by urushiols has the same molecular requirements as allergy induction, e.g., contact dermatitis, and the degree of allergenicity is dependent on the catecholic moiety and the number of double bonds present in the pentadecyl side chain (Fig. 5) [30, 31]. Notably, both structural features were required for urushiol-mediated inhibition of electron flow: the long aliphatic chain allows the insertion and trafficking of the allergen within biological membranes, and the catecholic moiety permits the covalent modification of self-proteins and enables the interaction with human CD1a [9]. Thus, our findings, together with our previous observation that urushiols can induce inflammation in SCID mice [7], suggest that these structures, which are similar to those of ubiquinone, might interfere with some mitochondrial functions, such as the production of the radicalary species that lead to cellular oxidative stress, generating danger signals that can trigger the stimulation of innate immunity [12, 14].

Ubiquinone and urushiols and their quinonic forms are extremely hydrophobic molecules; therefore, they are concentrated in the hydrophobic bilayer of membranes, making them good candidates as competitive inhibitors of electron flow particularly given their similarity with each other. Nonetheless, unexpectedly, the urushiol-mediated inhibition of electron flow was noncompetitive, suggesting that urushiols and ubiquinone do not share the same docking site at cytochrome b (Fig. 3). Moreover, our results show that the electron flow may be blocked because of urushiol mediation between b_L_ and b_H_, pointing to the discovery of a new docking site-specific to urushiols on cytochrome b. An alternative explanation is that the inhibition may be caused by the distortion of the cytochrome b structure [32] after the intercalation of the long aliphatic urushiol chain covalently bound to cyt c_1_ at lys 202, as discussed below.

The inhibition of the electron flow mediated by urushiols at the level of complex III plays an essential role in triggering the initiation of danger signaling [14, 26]. In this regard, it has been shown that antimycin A [33], a well-known complex III inhibitor, promotes the generation of free radicals [34] and superoxide, with the consequential stimulation of innate immunity, which subsequently stimulates the adaptive immune response. Furthermore, it has been demonstrated that ROS and superoxide induce the activation of NFκB, a master transcription factor that controls inflammation in innate immune cells [35]. Importantly, by stimulating NFκB, free radicals induce the expression of adhesion molecules on neighboring capillary vessels, which play important roles in lymphocyte homing [36, 37]. Moreover, NFκB induces the local production of a set of pro-inflammatory cytokines, including IL-1, IL-6, and TNF-α [38]. Due to the mitochondrial damage induced by urushiols, it is most likely that the transmembrane potential decreases, leading to the release of cytochrome c and other apoptogenic factors [39] from mitochondria, thereby inducing apoptosis of keratinocytes [40] and tumor cells lines [41].

The intense labeling of the tritiated litreol at cytochrome c_1_ (Figs. 6 and 7), as identified by tryptic fingerprinting (Additional file 1: Figure S3), was the most intriguing finding because it indicated that urushiols could modify mitochondrial self-proteins that, in turn, can be processed and presented to T lymphocytes.

Altogether, the modification of mitochondrial antigens by urushiols, the induction of ROS and apoptosis [36, 41] generate the perfect scenario for dendritic cell (DCs) maturation, including the expression of CD1a [9]. This process culminates with the migration of DCs to the draining lymph nodes and T cell activation [42]. The presentation of modified self-antigens from apoptotic cells by MHC class II molecules results in CD4^+^ T cell activation and the downregulation of the adaptive immune response; in contrast the presentation of modified self-antigens on class I molecules to CD8^+^ T cells promotes further inflammation, as described by Lopez et al. [7].

In the course of urushiol-induced allergy, mitochondrial antigens can undergo degradation by the mitophagy induced after organelle damage (Fig. 1), resulting in class II presentation to CD4^+^ T cells [43]. Moreover, mitochondria have a sophisticated and regulated proteolytic system to degrade misassembled or nonfunctional inner membrane proteins [44, 45]. The functional damage to cytochrome bc_1_ induced by urushiols, by chemical modification, superoxides or free radicals, as a result of the inhibited respiration chain may impair the binding of prohibitins, sensors of the structural integrity of mitochondrial proteins, which is a requisite to undergo partial degradation by AAA proteases located in the mitochondria. In this regard, peptides of more than 10 amino acid residues might be generated from the proteolytic degradation mediated by m-AAA in the matrix, which can be subsequently translocated by the MdL1-like transporter ABCB10 [46] into the intermembrane space [47]. Another pool of peptides can also be generated from proteolytic degradation by i-AAA in the intermembrane space. Once in the intermembrane space, peptides generated by i-AAA, as well as those generated by m-AAA, might subsequently diffuse into the cytosol through pores present in the outer membrane, as reviewed by Arnold and Langer [44]. Since the amino-terminal region of cytochrome c_1_ faces the intermembrane space, the self-peptides modified by urushiols should diffuse directly to the cytosol and subsequently bind the class I histocompatibility antigens in the endoplasmic reticulum. Under this paradigm, peptides derived from mitochondrial antigens have been described by Fischer-Lindahl et al. [48], on the cell surface in association with H2-M3 histocompatibility antigen.

Regarding the identification of the molecular targets of urushiols, in this study, we infer the generation of two modified peptides produced by the electrophilic modification of cytochrome c_1_ (Additional file 1: Figure S3). Since catecholic groups might form covalent adducts with lys or cys, it is tempting to speculate that lys 202 is one of the main targets of urushiols in cytochrome c_1_, which is in tight proximity with the transmembrane region [32]. The modification of lys 202 by urushiols could inhibit the electron flow at the level of cytochrome b because its long aliphatic chain may alter the structure of cytochrome b at a point critical for halting the transfer of electrons among the b cytochromes. Another possible explanation is that the modification of cytochrome c_1_ and the inhibition of cytochrome b might be two independent events. For this case, we postulate that urushiols interact noncovalently with cytochrome b, as described for most cytochrome b inhibitors [49], and similarly to the noncovalent interaction of the poison ivy urushiol with CD1a, as recently described by Kim et al. [9].

Alignment of the sequences available for cytochrome c_1_ from data banks (chicken, human, bovine, and mouse) [50–53] indicates that the hydrophobic transmembrane region and the region towards the amino-terminal sites of cytochrome c_1_ are conserved among mammals, suggesting that urushiols can similarly modify other mammalian cytochrome c_1_. However, since urushiols have a very long aliphatic chain, which is approximately 25 Å (Additional file 1: Figure S1), the transport of urushiol-modified peptides into the endoplasmic reticulum through TAP proteins involves an additional problem unless the aliphatic chain is shortened by ω-oxidation, as suggested by Kalergis et al. [16]. Nevertheless, it has been demonstrated that peptides displaying synthetic lateral chains with sizes similar to urushiols might be transported by the TAP system [51].

The inhibitory effect of urushiols at the level of mitochondrial respiration may represent a critical defense system in plants belonging to the *Anacardiaceae* family to avoid herbivorous predators and pathogenic fungi [54, 55]. It is noteworthy that *Rodentia*, being the order with the most mammalian species, including rats and mice, lacks CD1a and almost all group 2 lipid-presenting molecules [9, 10], suggesting an advantageous characteristic for them, perhaps expanding their sources of food.

In addition to gaining new insights into the molecular mechanisms of urushiol-induced dermatitis, this study shows urushiols as new and natural tools that may be evaluated for the development of better adjuvants because of the strong immunogenicity of urushiols and their capacity to induce pro-inflammatory signals via mitochondrial damage [41]. For instance, this urushiol-induced stimulation of the innate immune response might exert a bystander effect, as other non-specific immunostimulants [56], which could be used as an adjuvant in anti-tumoral immunotherapies to potentiate the specific immune response. In this context, a recent study has evaluated the use of a novel tumor-targeted urushiol-loaded micelle delivery system in a mouse model of breast cancer, showing that these micelles enhanced the accumulation of urushiol in the tumor and thus opening the possibility of their use in the treatment of breast cancer [57]. Concerning to the mechanisms involved in clinical allergy to urushiols in humans, in addition to the direct presentation of unmodified urushiols on CD1a molecules to human lymphocytes [9], our findings here suggest that there is also the generation of mitochondrial neoantigens, which would stimulate an specific T-cell response independently of CD1a. Finally, our data suggest an unexpected link between a mitochondrial protein and the induction of an allergic reaction. Thus, mitochondria may constitute a source of cellular targets for the generation of neoantigens involved in urushiol-induced allergies.

## Conclusions

Collectively, our results revealed that after epicutaneous painting with litreol on mouse ears, mitochondria underwent dramatic structural and functional alterations, showing that oxidative phosphorylation was affected. Furthermore, functional analyses demonstrated that electron flux was halted at the level of cytochromes b, more precisely in between b_L_ (cyt b_566_) and b_H_ (cyt b_562_). Subsequent proteomic analysis revealed that cytochrome c_1_ was the main molecular target of the nucleophilic attack mediated by litreol.

## Methods

### Chemicals

ADP, ascorbate, CCCP, duroquinol, ethylene glycol-bis (β-aminoethyl ether) *N*,*N*,*N*ʹ,*N*ʹ tetraacetic acid (EGTA), glutamate, 4-(2-hydroxy-ethyl)-l -piperazine-ethanesulfonic acid (HEPES), malate, succinate, *N*,*N*,*N*ʹ,*N*ʹ-tetramethyl-*p*-phenylenediamine (TMPD) and 3-methyl catechol were purchased from Sigma Chemical Co. (St. Louis, MO). Pentadecyl phenol was obtained from Cardolite Corporation (Bristol, PA USA). Duroquinol (Dur) was prepared from duroquinone as described previously [58]. Urushiols were dissolved in dimethyl sulfoxide (DMSO; Merck, Darmstadt, Germany). The solvent had no effect on respiration at any of the concentrations employed. All the other reagents were of the highest purity available.

### Allergens

Litreol, 3-(10-Z-pentadecenyl)-catechol, was obtained from the leaves of trees growing in the Olmué area (Chile) and purified by silica gel as described by Gambaro et al. [3]. Poison ivy/oak and pentadecyl catechol were generously provided by Dr. Alfred del Grosso from the FDA (USA). The structural formulas of the urushiols used in this study are shown in Additional file 1: Figure S1. ^3^H-litreol: (3-pentadecyl-(10,11-^3^H)-catechol was produced by ChemSyn Science Laboratories (USA) by catalytic reduction of cold litreol under tritium gas flux. The specific activity of the end product was 53.5 mCi/μM, and the tracer was maintained at − 40 °C in ethanol at a concentration of 200 μM until use.

### Animals

We used BALB/c female mice purchased from Biosonda S.A., (Santiago, Chile). Male Donryu rats of 180–230 g, which were starved for 24 h before the experiments, were from the Animal House Facility Facultad de Medicina, Instituto de Ciencias Biomédicas, Universidad de Chile. The study was performed in strict accordance with the Guidelines for the Care and Use of Laboratory Animals of the National Commission for Scientific and Technological Research of Chile Universidad de Chile. Bovine hearts were obtained from a 2-year-old female bovine at the Lo Valledor slaughterhouse (Santiago, Chile).

### Transmission electron microscopy

The ears of the BALB/c mice were painted with a 1% solution of litreol in chloroform or chloroform alone, as a control as described López et al. [7]. After 24 h, the ears were removed under general anesthesia, and the mice were euthanized. Small pieces of select regions of the ear skin from the control (exposed to solvent) and experimental (treated with litreol) mice were fixed for 6 h at room temperature in a solution containing 3% glutaraldehyde (Polyscience, USA), 100 mM KCl, 2 mM MgCl_2_, 0.25 M sucrose and 0.1 M PIPES buffer, pH 6.9. Then, these tissues were postfixed in 1% OsO_4_ in 0.1 M cacodylate buffer, dehydrated, and embedded in Epon (Polyscience) according to the method of Luft [59]. Thin sections were counterstained with uranyl acetate and lead citrate, according to Reynolds [60]. The preparations were examined and photographed at 80 kV on a JEOL 100-B electron microscope at 80 kV (Electronic Microscopy Facility, Pontificia Universidad Católica de Chile).

### Rat liver-isolated mitochondria

Mitochondrial suspensions of 50 mg protein/mL were prepared according to Pedersen et al. [61] with the following modifications: mitochondrial fractions were washed twice at 10,000×*g* for 10 min and resuspended in a minimal volume of homogenization medium in the absence of bovine serum albumin to avoid adsorption of hydrophobic molecules to serum albumin.

### Mitochondrial subfractionation

The mitochondrial subfractionation procedure was performed as described by Pavani et al. [62] and Pedersen et al. [61]. Briefly, the mitochondrial fraction was first treated with digitonin and then centrifuged at 15,000*g* for 10 min at 5 °C to obtain mitoplasts (pellet). Next, the supernatant was centrifuged at 144,000*g* for 40 min at 5 °C, and then, the pellet was resuspended in a small volume of buffer, which was composed of the outer membrane fraction (pellet) and its corresponding supernatant, the intermembrane fraction. The mitoplasts were washed twice and sonicated for four 30-s bursts at the maximum energy setting interspersed with 60-s cooling intervals. The resulting suspension was diluted with an equal volume of cold buffer and centrifuged at 15,000*g* for 10 min at 5 °C. The pellet was discarded, and the supernatant was centrifuged at 144,000*g* for 50 min at 5 °C. Finally, the pellet was resuspended in a small volume of isolation medium, with the supernatant being the matrix fraction and the pellet being the fraction enriched with the mitochondrial inner membrane (submitochondrial particles).

### Cytochrome reduction assays

The reduction in cytochromes b and c was assayed in 100 mM KH_2_PO_4_ (pH 7.4) containing 0.5 mg/ml submitochondrial particles and 0.05 mM cytochrome c. The reaction was initiated by adding 0.17 mM NADH and was monitored at 563–577 nm for cytochromes b, and 550–535 nm for cytochrome c [63] The time of each cycle was 20 s, and the reading was 0.5 s. NADH was added after cycle 9, and 10 µM litreol was added after cycle 18, as indicated by the respective arrows (Fig. 3a, b). In total, 50 cycles were completed. The reduction in cytochromes b_566–577_ and b_562–577_ was assayed in 100 mM KH_2_PO_4_ (pH 7.4), as previously described [22, 62], containing 0.1% dodecyl maltoside, in the presence of a specific inhibitor of complex III (5 µM antimycin A). The reaction was initiated by adding 20 µM duroquinol, either in the presence or in the absence of 10 µM litreol.

### Bovine heart-isolated mitochondria

Mitochondrial suspensions of 50 mg protein/mL were prepared from bovine hearts according to Ragan et al. [63], washed twice, and resuspended in a minimal volume of homogenization medium (components) in the absence of bovine serum albumin. Fresh mitochondrial preparations were used for the litreol binding experiments, and mitochondrial subfractionation of the outer membrane, inner membrane, and mitoplasts was performed according to Paradies et al. [34]. The protein concentration was determined by a modified Lowry reaction [64] and standardized with serum albumin.

### Assay of oxygen consumption

The rates of oxygen consumption were measured by polarography with a Clark oxygen electrode (Yellow Springs Instrument Co., Yellow Spring, OH, USA) and using a YSI model 53 monitor (Yellow Springs Instrument, Yellow Spring, OH, USA) linked to a 100 mV single-channel recorder [65]. The mitochondrial suspension was equilibrated for 2 min at 25 °C before the urushiols were added. All measurements were taken after a 2 min preincubation period with the allergen. These assays were performed at 25 °C as it is the typical temperature used for these determinations because greater temperatures implicate working with lesser oxygen concentration and higher mitochondrial damage. Moreover, temperatures higher than 25 °C involve stronger outer membrane impairment, lesser ΔΨ, and thereby enhanced H_2_O_2_ production.

### Incubation of isolated mitochondria with ^3^H-litreol

Mitochondria were adjusted to a final concentration equivalent to 1 mg/ml of protein. Then, as a tracer, ^3^H-litreol was added to the preparation and incubated for 30 min at 25 °C. Finally, cold litreol was added to a final concentration of 1 mM and incubated for 10 min to compete with the noncovalent radioactive tracer associated with membrane proteins.

### SDS-PAGE electrophoresis

The technique was performed as described by [66] in a gradient or linear version, with a separating gel from 10 to 20% polyacrylamide and 5% stacking gel. Protein samples were heated for 5 min at 100 °C in the presence of SDS and DTT or 2 β-mercaptoethanol (Merck, Germany). Gels were run at 80 V for 15 h at room temperature. Next, the gels were then fixed and stained with Coomassie Blue.

### 2D-PAGE

Samples containing inner membrane from bovine heart mitochondria were heated at 100 °C for 5 min in the presence of 1% SDS in 50 mM Tris–HCl (pH 6.8) and 1 mM EDTA, and then fractionated on a sephadex G-200 column equilibrated with 1% SDS in the buffer described above. The radioactive fraction was concentrated on a Centricon device, and 100 μg of protein was precipitated with 90% acetone and subjected to 2D electrophoresis as described by [24]. Briefly, in the first dimension, samples were separated by isoelectric points using an isoelectric focusing chamber (Bio-Rad, model 155). The pH 4–7 gradient was made in 3,9% acrylamide:bisacrylamide (30:1) gels in the presence of 9.17 M urea, 2% NP-40, 1.6% ampholytes with 4–6 pH range, 0.4% ampholytes with 3–10 pH range. Samples containing 100 μg protein were dissolved in lysis buffer (9.5 M urea, 2% NP-40, 1.6% ampholytes with 4–6 pH range, 0.4% ampholytes with 3–10 pH range, and 5% β-mercaptoethanol) and loaded in the first dimension gels. Isoelectric focusing was performed at 500 V for 18 h followed by 800 V for 1 h. To determine the pH gradient generated in the gels of the first dimension, the control gels were cut in 5 mm length pieces, incubated separately in ultra-pure water for 5 h. Then, the pH was determined in each segment. Afterwards, gels from the first dimension were incubated in 10% glycerol, 5% β-mercaptoethanol, 2.3% SDS and 62.5 mM Tris at pH 6.8 for 2 h. The second dimension was performed in 16% SDS-PAGE at 100 V for 16 h.

### Western blot analysis

A monospecific rabbit antiserum to bovine cytochrome c_1_ and a polyspecific sera to complex III were donated by Diego Gonzalez-Halphen [67] and used to identify cytochrome c_1_ and complex III on gels, respectively. The procedure of Towbin et al. [68] was used. Briefly, mitochondrial preparations or subfractions were run on SDS-PAGE gels as described above and then transferred to a 0.02 nitrocellulose membrane. The membrane was blocked with 2% skim milk in PBS and NaN_3_ for 2 h at room temperature and then incubated for 15 h at room temperature with antibodies diluted from 1/5,000 to 1/10,000 in blocking buffer. Next, the membranes were washed with PBS-Tween 0.02% and then incubated with a secondary antibody, anti-rabbit IgG conjugated to horseradish peroxidase (HRP, Pierce-Endogen, USA), and assayed with a NBT-BCIP system (Pierce, Endogen). The reaction was stopped with Milli-Q water.

### Photofluorography

To detect the radioactive proteins on fixed and stained polyacrylamide gels, the procedure previously described by Bonner et al. [69] was used with minor modification. The gels were soaked in 100% dimethyl sulfoxide (DMSO) twice for 30 min each time and then subjected to a PPO/DMSO procedure. In brief, the gels were impregnated with 2.5-diphenyl oxazole (PPO)-DMSO, rehydrated, dried between dialysis membranes, and exposed to Kodak X-OMAT film at − 70 °C for 24 h for unidimensional gels and 72 h for bidimensional gels. The densitometry analyses of the gels were performed using the Un-Scan-It Gel program (Orem, Utah, USA).

### Mass fingerprinting

Mass fingerprinting was performed at the Rockefeller University Protein Resource Center HHMI Biopolymer Facility. Slices of the 2D-PAGE containing radioactive spots were digested with trypsin, and the digested protein was subjected to mass analysis. The mass spectrum was compared with the information available in data banks.

### Statistical analysis

The results of the experiments are expressed as the mean ± SEM. Comparisons were made using a one-way analysis of variance (ANOVA) followed by Tukey’s test. All graphics and statistical analyses were performed using Sigma Stat 2.01 (SPSS., Chicago IL, USA). Significance was defined at a p-value ≤ 0.05.

## Supplementary Information

**Additional file 1: Figure S1.** Structure of catecholic and non-catecholic compounds. **Figure S2.** Analysis of the potential aminoacids targets of litreol in the cytochrome c_1_. **Figure S3.** Litreol alters the mass fingerprinting, as indicated by the tryptic fragments from the radioactive spots of the 2D-PAGE gel analyzed by mass spectrometry and compared with the native cytochrome c_1_.

## Data Availability

The data that support the findings of this study are available from the corresponding author upon reasonable request.

## Abbreviations

Ant: Antimycin
CCCP: Carbonyl cyanide *m*-chlorophenylhydrazone
DMSO: Dimethyl sulfoxide
Dur: Duroquinol
lys: Lysine
NADH: Reduced nicotinamide adenine dinucleotide
NF-kB: Nuclear factor k-B
PBS: Phosphate-buffered saline
SCID: Severe combined immunodeficiency
ROS: Reactive oxygen species
SDS-PAGE: Sodium dodecyl sulfate-polyacrylamide gel electrophoresis
TAP: Transporter-associated proteins
2D-PAGE: Two-dimensional gel electrophoresis
TMPD: *N*,*N*,*N*ʹ,*N*ʹ-Tetramethyl-*p*-phenylenediamine
QH2: Ubiquinol
