# Supplementary material for: Allergens of the urushiol family promote mitochondrial dysfunction by inhibiting the electron transport at the level of cytochromes b and chemically modify cytochrome c1
Source: Biol Res. 2021 Oct 28;54:35. doi: 10.1186/s40659-021-00357-z (PMC8554850; doi:10.1186/s40659-021-00357-z)
Supplement: Supplementary file 1 — Additional file 1: Figure S1. Structure of catecholic and non-catecholic compounds. Figure S2. Analysis of the potential aminoacids targets of litreol in the cytochrome c1. Figure S3. Litreol alters the mass fingerprinting, as indicated by the tryptic fragments from the radioactive spots of the 2D-PAGE gel analyzed by mass spectrometry and compared with the native cytochrome c1. [file 40659_2021_357_MOESM1_ESM.pdf]

ADDITIONAL FILE 1

Allergens of the urushiol family promote mitochondrial dysfunction by inhibiting the electron transport at the level of cytochromes b and chemically modify cytochrome c_1

Rodrigo Pacheco, Sergio A. Quezada, Alexis M. Kalergis, María Inés Becker, Jorge Ferreira and Alfredo E De Ioannes

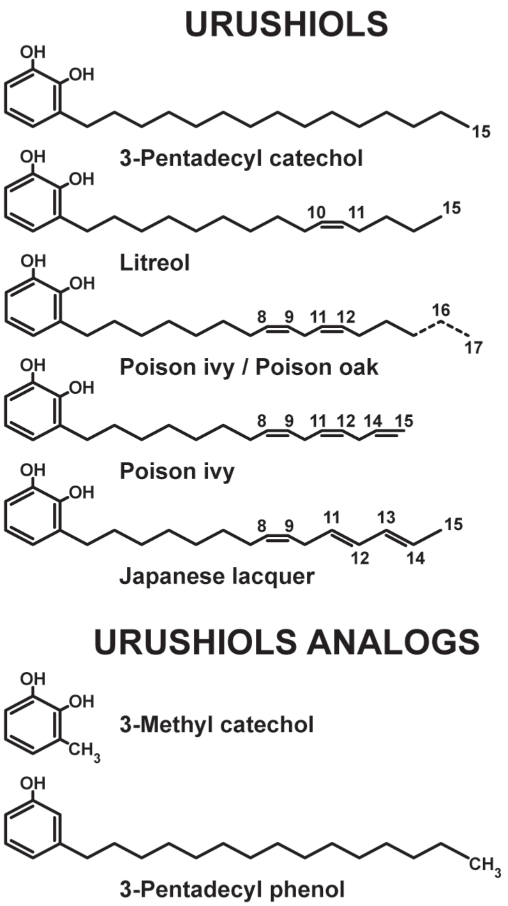

Figure S1. **Structure of catecholic and non-catecholic compounds.**

1 SDLELHPPSY PWSHRGLLSS LDHTSIRRGF QVYKQVCSSC HSMDYVAYRH

51 LVGVCYTEDE AKALAEEVEV QDGPNEDGEM FMRPGKLSDY FPKPYPNPEA

101 ARAANNGALP PDLSYIVRAR HGGEDYVFSL LTGYCEPPTG VSLREGLYFN

151 PYFPGQAIGM APPIYNEVLE FDDGTPATMS QVAKDVCTFL RWAAEPEHDH

201 RKRMGL**KMLL** **MMGLLLPLVY** **AMK**RHKWSVL KSRKLAYRPP K

Figure S2. Analysis of the potential aminoacids targets of litreol in the cytochrome c1. The aminoacid sequence of the bovine cytochrome c1 obtained by Iwata et al [52] is shown. The α-helix in the C-terminal side is indicated in red. Aminoacids located inside the inner membrane are indicated in bold. Lysines commented in the text are underlined. Green boxes indicate the sequence of peptides identified in the mass fingerprinting that disappear from the analysis upon treatment with litreol (see figure S3).

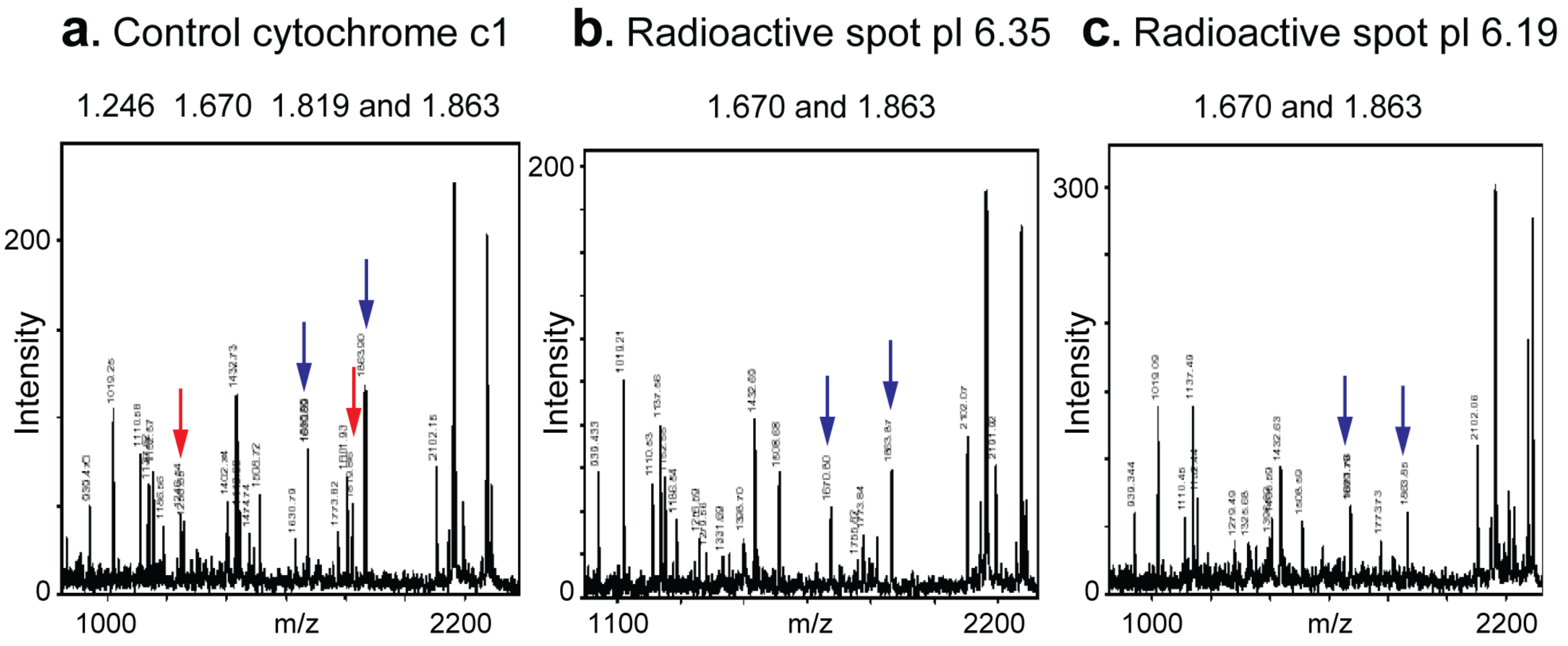

Figure S3. Litreol alters the mass fingerprinting, as indicated by the tryptic fragments from the radioactive spots of the 2D-PAGE gel analyzed by mass spectrometry and compared with the native cytochrome c_1. A) Unmodified cytochrome c1. **b**) and **c**) Radioactive spots corresponding to a pI of 6. 35 and 6.19, respectively. The m/z of the tryptic fragments of identical size from the predicted fragments obtained from cytochrome c_1 are indicated with arrows. Fragments shown in red arrows are 1-15 (1.819) and 192-202 (1.246) that, after treatment with litreol, were not observed because they were out of the range for detection by the instrument.
